# Complete Genome Sequence of *Escherichia coli* Phage vB_EcoM_Alf5

**DOI:** 10.1128/genomeA.00315-17

**Published:** 2017-05-18

**Authors:** Laurynas Alijošius, Eugenijus Šimoliūnas, Laura Kaliniene, Rolandas Meškys, Lidija Truncaitė

**Affiliations:** Department of Molecular Microbiology and Biotechnology, Institute of Biochemistry, Life Sciences Center, Vilnius University, Vilnius, Lithuania

## Abstract

Here, we announce the complete genome sequence of the *Escherichia coli* myophage vB_EcoM_Alf5 belonging to the genus *Felixo1virus*, whose members have not been comprehensively studied at the molecular level. Phage vB_EcoM_Alf5 infects *E. coli* K-12-derived laboratory strains and therefore is well suited for functional studies.

## GENOME ANNOUNCEMENT

Pathogenic *Escherichia coli* and *Salmonella* strains are the major causative agents of foodborne diseases in both humans and animals. With the emergence of multidrug-resistant strains, bacteriophages have been proposed as an alternative antimicrobial (1–7). A number of bacteriophages potentially suitable for the biocontrol of *E. coli* and *Salmonella* spp. have been isolated and sequenced, but only a few of them have been functionally characterized at the molecular level. In this study, we describe the complete genome sequence of the *E. coli*-specific myophage vB_EcoM_Alf5 (Alf5) capable of infecting *E. coli* K-12-derived laboratory strains, including KEIO collection mutants (8).

Bacteriophage Alf5 was isolated from water samples collected from the Nemunas River in Lithuania using *E. coli* MH1 strain as a host. Transmission electron micrographs of phage particles showed that Alf5 virion morphology was indistinguishable from that of other *Felixo1virus* phages (9–11). The isolated genomic DNA of Alf5 was sequenced at BaseClear group in the Netherlands using Illumina HiSeq 2500 DNA sequencing technology. *De novo* assembly of the genome sequence data was carried out using CLC Genomics Workbench software version 8.5.1. The assembled contigs were then scaffolded using the SSPACE Premium scaffolder version 2.3, which resulted in a single scaffold of 87,662 bp in length (2,975-fold coverage), with a G+C content of 39.02%.

The NCBI BLASTn tool was used to perform the whole-genome sequence alignments and subsequent phylogenetic analysis. The open reading frames (ORFs) in the Alf5 genome were predicted with Glimmer version 2.02 (12) and Geneious version 5.5.6 (13) and annotated based on the results of BLASTp analysis. tRNAscan-SE 1.21 (14) was used to identify tRNAs.

BLASTn analysis revealed that the genome sequence of Alf5 is most similar to that of felixo1virus vB_EcoM-VpaE1 (VpaE1) (93% coverage, 96% identity), which infects *E. coli* B strains with an incomplete core lipopolysaccharide (11). The restriction digestion analysis *in silico* using Restriction Mapper version 3 tool (http://www.restrictionmapper.org) showed that the Alf5 genome sequence contains no recognition sites for many restriction enzymes, including BamHI, XhoI, SacI, PstI, and even MboI, which recognizes GATC, a trait also observed in other phages of the same genus (11).

The genome of Alf5 has a total of 133 ORFs and 19 tRNAs. A large number of encoded tRNAs and homing endonucleases (3 in Alf5), as well as the presence of genes for RIIA, RIIB, lysin, and glutaredoxin, is a characteristic feature of *Felixo1virus* phages. With the exception of 2 putative tail fiber proteins (gp73 and gp74), the predicted Alf5 structural proteins, as well as those involved in metabolism of nucleic acids, are most closely related to their counterparts found in bacteriophages VpaE1 and Felix 01 (86 to 100% identity). The most obvious differences between the genomes of Alf5 and other *Felixo1virus* phages are observed in the ORFs, which encode hypothetical and tail fiber proteins, possibly accounting for their host range.

In conclusion, bacteriophage Alf5 is a good model for both analysis of the differences in the host ranges and functional characterization of genetically related *Felixo1virus* phages.

### Accession number(s).

The complete genome sequence of vB_EcoM_Alf5 was deposited in GenBank under the accession number KX377933.
